# Acute Kidney Injury Induces Oxidative Stress and Hepatic Lipid Accumulation through AMPK Signaling Pathway

**DOI:** 10.3390/antiox12040883

**Published:** 2023-04-05

**Authors:** Kathy K. W. Au-Yeung, Yue Shang, Charith U. B. Wijerathne, Susara Madduma Hewage, Yaw L. Siow, Karmin O

**Affiliations:** 1 St. Boniface Hospital Research Centre, Winnipeg, MB R2H 2A6, Canada; 2 Department of Animal Science, University of Manitoba, Winnipeg, MB R3T 2N2, Canada; 3 Department of Physiology & Pathophysiology, University of Manitoba, Winnipeg, MB R3E 0J9, Canada; 4 Agriculture and Agri-Food Canada, St. Boniface Hospital Research Centre, Winnipeg, MB R2H 2A6, Canada

**Keywords:** AKI, AMPK, lipid, fatty liver, oxidative stress

## Abstract

Acute kidney injury (AKI) often impairs the function of other organs leading to distant organ injury. The liver is the major organ that regulates metabolism and lipid homeostasis in the body. It has been reported that AKI causes liver injury with increased oxidative stress, inflammatory response and steatosis. In the present study, we investigated the mechanisms by which ischemia-reperfusion-induced AKI caused hepatic lipid accumulation. Kidney ischemia (45 min)-reperfusion (24 h) led to a significant increase in plasma creatinine and transaminase in Sprague Dawley rats, indicating kidney and liver injury. Histological and biochemical analyses revealed hepatic lipid accumulation with a significant elevation of triglyceride and cholesterol levels in the liver. This was accompanied by a decreased AMP-activated protein kinase (AMPK) phosphorylation, indicating the reduced activation of AMPK, which is an energy sensor that regulates lipid metabolism. The expression of AMPK-regulated genes that were responsible for fatty acid oxidation (CPTIα, ACOX) was significantly decreased, while the expression of lipogenesis genes (SREPB-1c, ACC1) was significantly elevated. The oxidative stress biomarker malondialdehyde was elevated in the plasma and liver. Incubation of HepG2 cells with an oxidative stress inducer hydrogen peroxide inhibited AMPK phosphorylation and caused cellular lipid accumulation. This was accompanied by decreased expression of genes responsible for fatty acid oxidation and increased expression of genes responsible for lipogenesis. These results suggest that AKI elicits hepatic lipid accumulation through decreased fatty acid metabolism and increased lipogenesis. Oxidative stress may contribute, in part, to the downregulation of the AMPK signaling pathway leading to hepatic lipid accumulation and injury.

## 1. Introduction

Acute kidney injury (AKI) often occurs as a complication after major surgeries or in critically ill patients. Ischemia-reperfusion is one of the most common risk factors causing AKI. It is characterized by acute renal failure and can have negative impacts on the outcomes of hospitalized patients [1,2]. Despite the availability of renal replacement therapy, AKI is associated with high mortality, increased hospital stay and healthcare costs [3]. The crosstalk between organs was recognized to play a crucial role in deciding the therapeutic regimen for critically ill patients to reduce mortality [4]. Over the past decade, there has been extensive research into the mechanisms of distant organ dysfunction caused by AKI, in which the most studied organ was the lung, followed by the heart, brain, liver, and gut [4,5,6]. AKI patients with a complication of liver dysfunction had poor clinical outcomes [7,8]. Several clinical studies reported that AKI patients with hepatic dysfunction had alterations in protein synthesis, inflammatory responses, and abnormal metabolism of lipids, proteins, and drugs [5,9]. AKI-induced liver injury in experimental animals led to increased oxidative stress, hepatocyte necrosis, elevated cytokine levels, and infiltration of leukocytes [4,10]. The effect of liver disease on kidney damage is well-studied. However, the impact of AKI on hepatic dysfunction and lipid accumulation remains unclear.

The liver has a pivotal role in the synthesis and degradation of lipids and the oxidation of fatty acids. Fat can either be stored as triglyceride in lipid droplets or used to produce phospholipids in the liver [11]. Excess dietary glucose can be converted into fat in hepatocytes through the process of lipogenesis. When the elevation of fatty acid exceeds the capacity of fatty acid oxidation, triglyceride synthesis increases, and hepatic lipid accumulation occurs. This process, known as hepatosteatosis, is a hallmark of both alcoholic and nonalcoholic fatty liver disease (NAFLD) [12]. The AMP-activated protein kinase (AMPK) pathway is a vital cellular energy metabolic switch involved in the regulation of lipid metabolism in the liver and is regarded as a therapeutic target of NAFLD [13]. AMPK is activated by the phosphorylation of Thr-172 on the AMPKα catalytic subunit [14] but may also be regulated by oxidative stress [15]. In the context of AKI, changes in AMPK signaling can have important implications for the metabolic status of the kidney and other organs. During renal ischemia-reperfusion (IR), a depletion of intracellular ATP and a corresponding increase in intracellular AMP levels can lead to changes in AMPK activity [16]. In contrast, the blocking of AMPK activation may alter both anabolic pathways (lipid synthesis) and catabolic pathways (fatty acid oxidation) [17]. Although there is a correlation between AMPK downregulation and fatty liver, little information is available regarding the role of AMPK signaling in hepatic lipid accumulation in the context of AKI.

The metabolism of lipids is modulated by various enzymes that are involved in lipogenesis and fatty acid oxidation [18]. The expressions of these enzymes are regulated by transcription factors such as sterol regulatory element-binding protein-1 (SREBP-1c) and peroxisome proliferator-activated receptors (PPARs) in the liver. In NAFLD, an accumulation of fatty acids in hepatocytes leads to an excess burden on mitochondria and an increase in the production of reactive oxygen species (ROS). These ROS can promote the modification of macromolecules, including lipid peroxidation, and compromise the antioxidant defense system [19,20]. It was reported that hydrogen peroxide could enhance SREBP-1c expression, leading to an increase in fatty acid synthesis and promoting lipogenesis [21]. Studies also showed that antioxidants could attenuate hepatic steatosis through the downregulation of SREBP-1c expression [22,23]. Our recent study demonstrated that liver function was impaired in rats with renal IR, in which hepatic glutathione production was inhibited, leading to oxidative stress [10]. It is plausible that AKI-induced oxidative stress may, in part, be responsible for the alterations in lipid metabolism and homeostasis.

The liver is the main organ for regulating lipid metabolism and homeostasis in the body [24]. It was reported that activation of AMPK and phosphorylation of acetyl-CoA carboxylase-1 (ACC1) by AMPK-activating drugs were crucial for the improvement of fatty liver disease [17,25,26]. Our recent studies showed that renal IR-induced oxidative stress contributed to impaired liver function and systemic inflammation in rats [10,27]. We also found that attenuation of oxidative stress by lingonberry supplementation could activate AMPK and improve high-fat diet-induced fatty liver injury [28]. Another study reported that a distant effect of IR-induced AKI caused hepatic steatosis and liver injury in mice [29]. In the present study, we investigated the effect of kidney IR on hepatic lipid metabolism and the interaction between oxidative stress and AMPK signaling.

## 2. Materials and Methods

### 2.1. Induction of Renal Ischemia-Reperfusion in Rats

Sprague Dawley rats (male, 250–300 g) were purchased from the University of Manitoba Central Animal Care Services (Winnipeg, MB, Canada). The rats were randomly divided into two groups: (1) sham-operated control (Sham) and (2) kidney IR. To induce AKI, the left renal pedicle was clamped for 45 min with a non-traumatic vascular clamp to induce renal ischemia in the IR group. After 45 min, the clamp was removed to allow blood flow to the left kidney (reperfusion), and a right nephrectomy was performed following the protocol outlined in previous studies [27,30]. The Sham group underwent the same surgical procedure but without kidney ischemia and were sacrificed 24 h after the surgery. At the end of the experimental period, the liver and blood were collected. Plasma was prepared, and creatinine, aspartate transaminase (AST), and alanine transaminase (ALT) levels were measured using the Cobas C111 Analyzer (Roche, Laval, QC, Canada). The study was conducted in accordance with the Guide to the Care and Use of Experimental Animals published by the Canadian Council on Animal Care, and the University of Manitoba Protocol Management and Review Committee approved all procedures.

### 2.2. Cell Culture

The human hepatoma cell line (HepG2, cell line: HB-8065) was purchased from the American Type Culture Collection (Manassas, VA, USA) and cultured in Dulbecco’s modified eagle medium (DMEM) (VWR, Radnor, PA, USA) supplemented with 10% fetal bovine serum (Hyclone Laboratories Inc., Logan, UT, USA) under a humidified atmosphere with 5% CO_2_ at 37 °C. The cells were cultured in 96-well plates, 6-well plates, or 60 mm dishes at a density of 1 × 10^5^ cells/mL. After overnight incubation, cells were treated with hydrogen peroxide (H_2_O_2_) for 6, 12, or 24 h, and subsequent assays were carried out. H_2_O_2_ was used to induce oxidative stress in HepG2 cells. To examine cell viability, water-soluble tetrazolium salt (WST-1, Sigma-Aldrich, Taufkirchen, Germany) was used according to the manufacturer’s instructions. Briefly, HepG2 cells were cultured overnight in 96-well plates and then exposed to various concentrations of H_2_O_2_ ranging from 0 to 500 µM for 24 h. The cells were washed twice with phosphate-buffered saline (PBS), and 100 µL of culture medium containing 10% WST-1 was added to each well. After 1 h of incubation, the absorbance was measured at 450 nm using a microplate reader (Molecular Devices, San Jose, CA, USA). Cell viability was expressed as the percentage of untreated control cells (0 h).

### 2.3. Biochemical Analysis

Malondialdehyde (MDA) is a biomarker of lipid peroxidation. The MDA was measured in the liver and plasma using the thiobarbituric acid reactive substances (TBARS) method [10,31]. In brief, liver or cell samples were homogenized in PBS. Tissue and cell extracts were incubated with 10% phosphotungstic acid for 10 min and then centrifuged at 1000× *g*. The supernatants were collected and incubated with 0.67% thiobarbituric acid at 95 °C for 1 h. The absorbance was measured at 532 nm. The concentration of MDA was calculated and adjusted with protein concentration. Hepatic lipids were extracted from the liver tissue using the Folch method [32]. Briefly, the liver tissue was homogenized using a solution consisting of chloroform, methanol, and distilled water (in a v:v:v ratio of 4:2:3). The resulting homogenate was centrifuged to obtain the lipid-soluble phase, which was then dried using nitrogen gas. The dried lipids were subsequently dissolved in ethanol. To measure intracellular triglycerides, cells were collected in PBS and then sonicated as previously described [33]. Triglyceride and total cholesterol in the liver extract as well as the intracellular triglyceride, were measured using commercial kits according to the manufacturer’s instructions (Sekisui Diagnostics, Burlington, MA, USA).

### 2.4. Histological Staining

A portion of the liver tissue was fixed in 10% neutral-buffered formalin and embedded in paraffin. The paraffin-embedded liver tissue was sectioned into 5 μm thickness and stained with hematoxylin and eosin (H&E) for assessing morphological changes. The cryofixed liver tissue (10 μm) was stained with Oil Red O to evaluate lipid accumulation. The cells were also stained with Oil Red O to visualize neutral lipid accumulation as previously described [33].

### 2.5. Quantitative Real-Time PCR

The relative mRNA expression of acetyl-CoA carboxylase-1 (ACC1), acyl-CoA oxidase1 (ACOX), carnitine palmitoyltransferase-I-alpha (CPTIα), sterol regulatory element-binding protein-1 (SREBP-1c) and actin was determined by quantitative real-time polymerase chain reaction (qPCR) analysis using the StepOnePlus^TM^ Real-Time PCR System (Applied Biosystems, Foster City, CA, USA). In brief, total RNAs were extracted from the liver tissue using Trizol reagent (Invitrogen, Carlsbad, CA, USA), and cDNA was prepared as previously described [33]. The qPCR mixture was prepared by mixing cDNA (100 ng), 1X iTaq Universal SYBR Green Supermix (300 nM per primer, Bio-Rad, Hercules, CA, USA) and RNase-free water in a total reaction mixture of 20 μL. The comparative CT method was used to analyze the data, and the gene expression levels were normalized to that of the housekeeping gene (β-actin). The primer sequences used in this study are presented in Table 1.

### 2.6. Western Immunoblotting Analysis

Total proteins were extracted from either rat liver tissue or cells in a lysis buffer. The quantified proteins were separated by electrophoresis in a 10% SDS polyacrylamide gel, as previously described [10,28]. The proteins were subsequently transferred from the gel onto a nitrocellulose membrane. To determine the relative amount of pAMPK, the membranes were probed with rabbit anti-phospho-AMPK-a monoclonal antibody (1:1000) or rabbit anti-AMPK-a monoclonal antibody (1:1000) that were purchased from Cell Signaling Technology (Danvers, MA, USA). To ensure equal loading of protein quantity in each lane of the gel, the membranes were probed with an antibody against a housekeeping protein, rabbit anti-β-actin monoclonal antibody (1:1000, Cell Signaling Technology). HRP-conjugated anti-rabbit IgG secondary antibodies (1:2000, Cell Signaling Technology, Danvers, MA, USA) were used, and the protein bands were detected using the Enhanced Chemiluminescence detection system Millipore Ltd., Burlington, MA, USA). The amount of protein in each lane was then quantified using Quantity One software version 4.6.8 for Windows (Bio-Rad, Herculesm, CA, USA).

### 2.7. Statistical Analysis

The statistical analysis was carried out using GraphPad Prism 8 for windows (Version 8.0.2). Results were analyzed using an unpaired 2-tailed *t*-test. All the data were expressed as the mean ± SE. A statistical significance was considered when the *p*-value was less than 0.05.

## 3. Results

### 3.1. Renal Ischemia-Reperfusion Impaired Kidney and Liver Function

**Figure 1:**
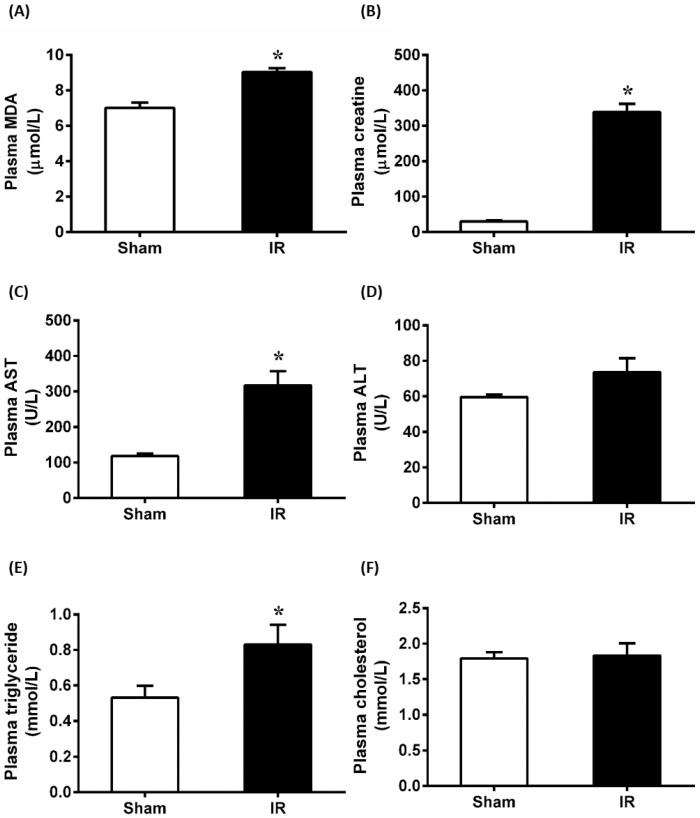
Renal ischemia-reperfusion induced kidney and liver injury. Rats were subjected to kidney ischemia-reperfusion (IR) or sham operation. Plasma levels of (**A**) malondialdehyde (MDA), (**B**) creatinine, (**C**) aspartate transaminase (AST), (**D**) alanine transaminase (ALT), (**E**) triglyceride, and (**F**) cholesterol were measured. Results are expressed as mean ± SE (*n* = 6–8). * *p* < 0.05 when compared with the value obtained from the Sham group.

Renal ischemia for 45 min followed by reperfusion for 24 h caused a significant elevation of plasma MDA and creatinine, indicating that IR caused oxidative stress and impaired kidney function (Figure 1A,B). At 24 h after renal IR, there was a significant increase in plasma AST level, while ALT levels remained unchanged in rats as compared to Sham (Figure 1C,D). Additionally, renal IR resulted in a significant increase in plasma triglyceride levels, but no change in cholesterol level was observed (Figure 1E,F). These findings suggested that renal IR not only caused kidney damage but also impaired liver function and increased hepatic lipid levels.

### 3.2. Renal Ischemia-Reperfusion Caused Oxidative Stress and Increased Lipid Level in the Liver

**Figure 2:**
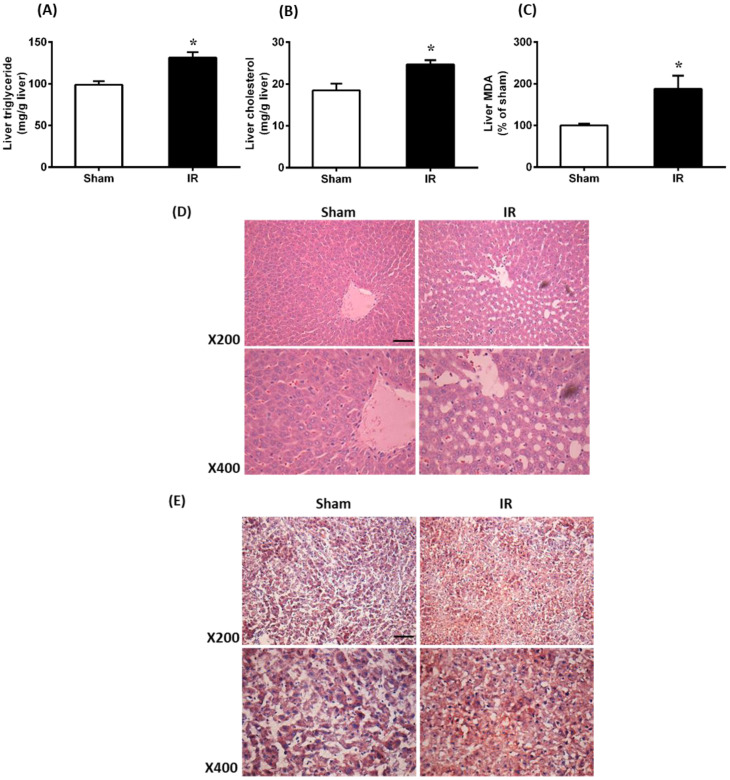
Effect of renal ischemia-reperfusion on lipid and malondialdehyde levels, and histological changes in the liver. Rats were subjected to kidney ischemia-reperfusion (IR) or sham operation. Liver (**A**) triglyceride, (**B**) cholesterol and (**C**) malondialdehyde (MDA) levels were measured. The MDA concentration in Sham rats was 0.56 nmol/mg protein. Results are expressed as mean ± SE (*n* = 6–8). * *p* < 0.05 when compared with the value obtained from Sham. (**D**) Paraffin sections of the liver tissue were stained with hematoxylin and eosin (H&E) to examine the histological changes. Representative hematoxylin and eosin (H&E) staining images of liver sections are shown (Scale bar = 100 μm, magnification ×200, ×400). (**E**) Liver tissues were stained with Oil Red O staining for neutral lipids.

To investigate whether AKI affected lipid levels in the liver, the levels of triglyceride and cholesterol were examined in the liver samples. Results showed that renal IR resulted in significantly higher triglyceride and cholesterol levels in the liver compared to Sham (Figure 2A,B). Renal IR caused a significant elevation of MDA in the liver, indicating increased lipid peroxidation (Figure 2C). Additionally, the gross appearance of liver tissue was examined by hematoxylin and eosin (H&E) staining and analyzed at both ×200 and ×400 magnification. The liver of the IR group showed vacuolization when compared to the Sham group (Figure 2D). Liver tissue was also stained with Oil Red O to examine lipid droplets. There were increased lipid vacuoles/droplets in the liver tissue of rats with renal IR compared to the Sham group (Figure 2E). Taken together, renal IR caused oxidative stress and lipid accumulation in the liver.

### 3.3. Renal Ischemia-Reperfusion Downregulated AMPK Pathway and Affected Lipid Metabolism in the Liver

**Figure 3:**
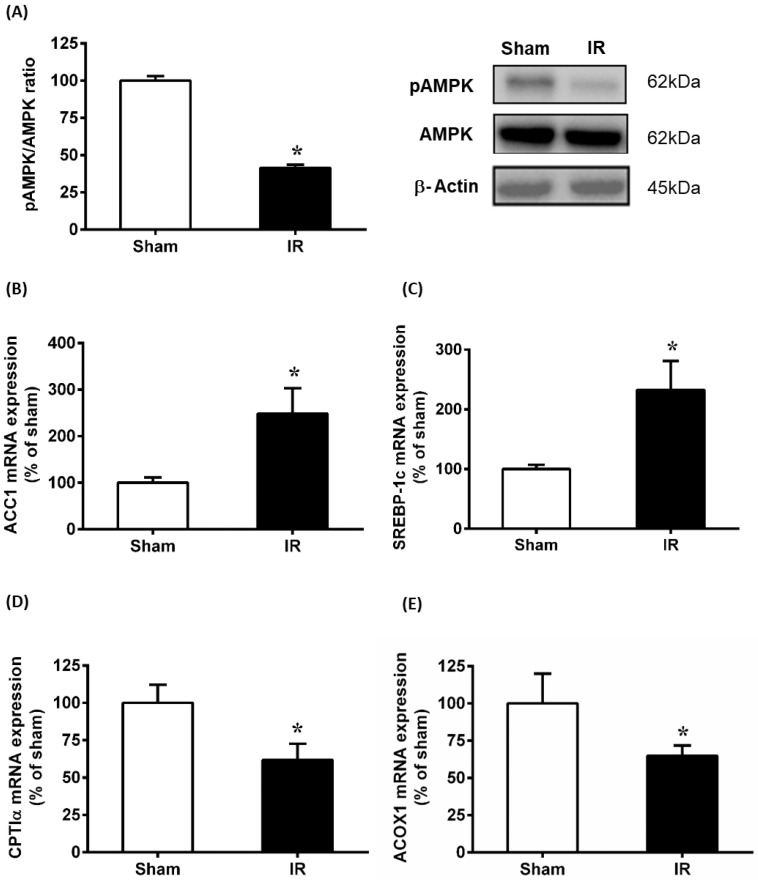
Effect of renal ischemia-reperfusion on the expression of hepatic AMPK and lipid metabolizing enzymes. Rats were subjected to kidney ischemia-reperfusion (IR) or sham operation. (**A**) Liver phosphorylated AMPK and total AMPK were determined by Western immunoblotting analysis. The mRNA of (**B**) ACC1, (**C**) SREBP-1c, (**D**) CPTIα and (**E**) ACOX were measured in the liver. The results are expressed as the means ± SE (n = 6 to 8). * *p* < 0.05 when compared with the value obtained from Sham.

AMPK is regarded as a therapeutic target for ischemia-reperfusion injury [34]. In the present study, the phosphorylation of AMPK was assessed by detecting pAMPK protein relative to total AMPK protein levels in the liver. Results showed that renal IR caused a significant decrease in the pAMPK protein level (Figure 3A). To investigate if lipid metabolism was affected by the downregulation of AMPK, genes involved in lipogenesis and fatty acid oxidation were examined. Results showed that renal IR significantly elevated ACC1 and SREBP-1c mRNA expression in the liver (Figure 3B,C). In addition, genes regulating fatty acid oxidation (ACOX1 and CPTIα) were decreased markedly (Figure 3D,E). Taken together, renal IR downregulated the phosphorylation of AMPK, which might lead to the alteration of lipid metabolism and subsequent lipid accumulation in the liver.

### 3.4. Effect of Hydrogen Peroxide (H_2_O_2_) on Lipid Accumulation in HepG2 Cells

**Figure 4:**
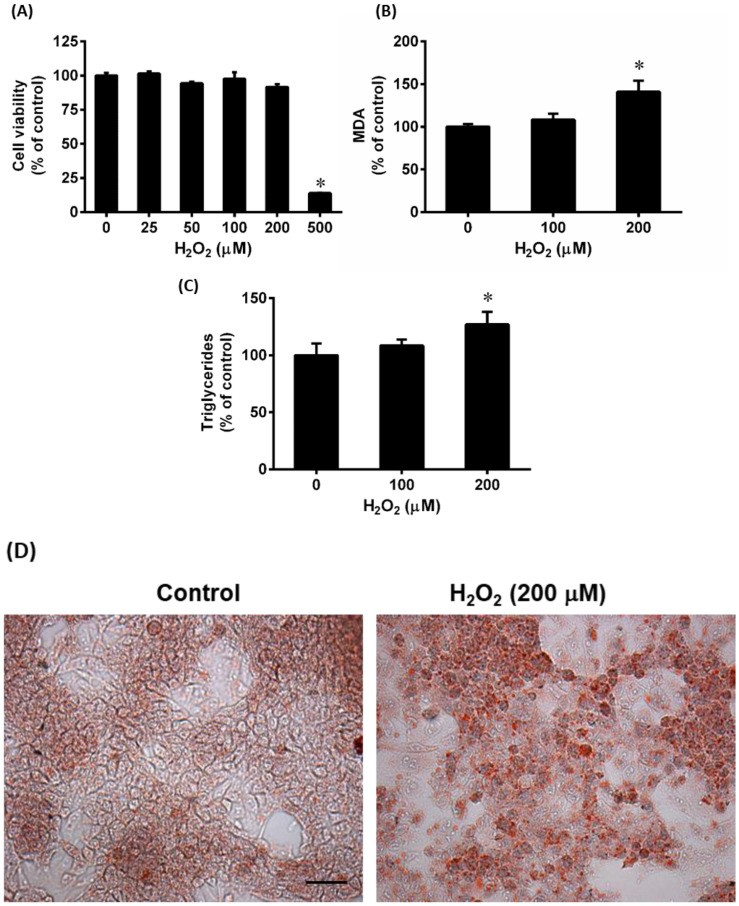
Effects of hydrogen peroxide on cell viability and lipid accumulation in HepG2 cells. Cells were incubated with hydrogen peroxide (H_2_O_2_) ranging from 25 to 500 μM for 24 h, and cell viability was examined (**A**). Results were expressed as a percentage of untreated control cells (0 h). Separate sets of cells were incubated with H_2_O_2_ and used to measure intracellular (**B**) malondialdehyde (MDA) and (**C**) triglyceride levels. The MDA and triglyceride concentrations in the control cells were 0.71 nmol/mg protein and 1.32 mmol/g protein, respectively. The results are expressed as means ± SE (*n* = 4 to 6). * *p* < 0.05 when compared with the value obtained from the control group. Another set of cells was stained with (**D**) Oil Red O to visualize intracellular lipids (Scale bar = 100 μm, magnification = 400×).

To induce oxidative stress, HepG2 cells were challenged with H_2_O_2,_ which is a widely used agent to cause oxidative stress in cellular models [35]. After cells were treated with various concentrations of H_2_O_2_, no significant toxicity was observed up to 200 μM of H_2_O_2_ (Figure 4A). Treatment of cells with H_2_O_2_ (0, 100, and 200 μM) for 24 h resulted in a significant increase in MDA level compared to control cells (Figure 4B). Additionally, the intracellular triglyceride level increased significantly (Figure 4C). To visualize intracellular lipids, cells were stained with Oil Red O. Cells incubated with H_2_O_2_ (200 μM) displayed much more red-stained lipid droplets compared to the control cells (Figure 4D). These results indicate that H_2_O_2_-induced oxidative stress caused lipid accumulation in HepG2 cells.

### 3.5. Effect of Hydrogen Peroxide (H_2_O_2_) on AMPK Phosphorylation and Lipid Metabolizing Gene Expression

**Figure 5:**
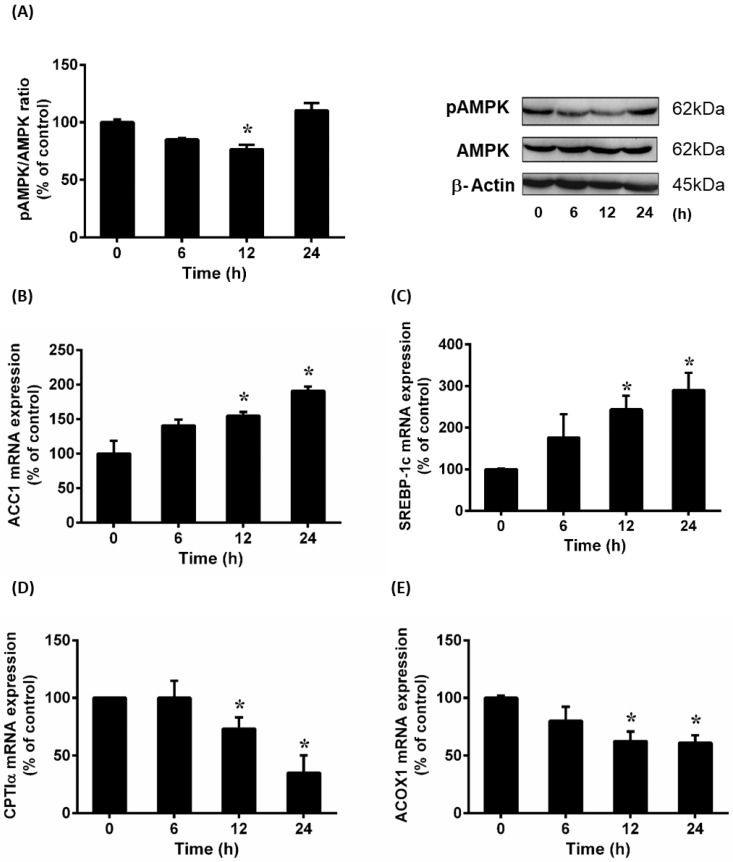
Effect of hydrogen peroxide on the expression of AMPK and lipid metabolizing enzymes in HepG2 cells. Cells were incubated with hydrogen peroxide (H_2_O_2_) for various time periods. (**A**) Phosphorylated AMPK and total AMPK proteins were determined by Western immunoblotting analysis. Relative mRNA expression of (**B**) ACC1, (**C**) SREBP-1c, (**D**) CPTIα and (**E**) ACOX1 were measured by real-time PCR analysis. The results are expressed as means ± SE (*n* = 4 to 6). * *p* < 0.05 when compared with the value obtained from the control group (0 h).

To further investigate the effect of H_2_O_2_ on lipid metabolism in HepG2 cells, AMPK phosphorylation status was examined. There was a significant decrease in the level of phosphorylated AMPK protein in cells incubated with H_2_O_2_ (Figure 5A). Incubation of cells with H_2_O_2_ caused a significant increase in the expression of lipogenesis genes ACC1 (Figure 5B) and SREBP-1c (Figure 5C). On the other hand, incubation of cells with H_2_O_2_ resulted in a significant decrease in mRNA expression of the enzymes (ACOX1 and CPTIα) that were involved in fatty acid oxidation (Figure 5D,E). These results suggested that oxidative stress could alter AMPK phosphorylation and lipid metabolizing enzyme expression.

## 4. Discussion

**Figure 6:**
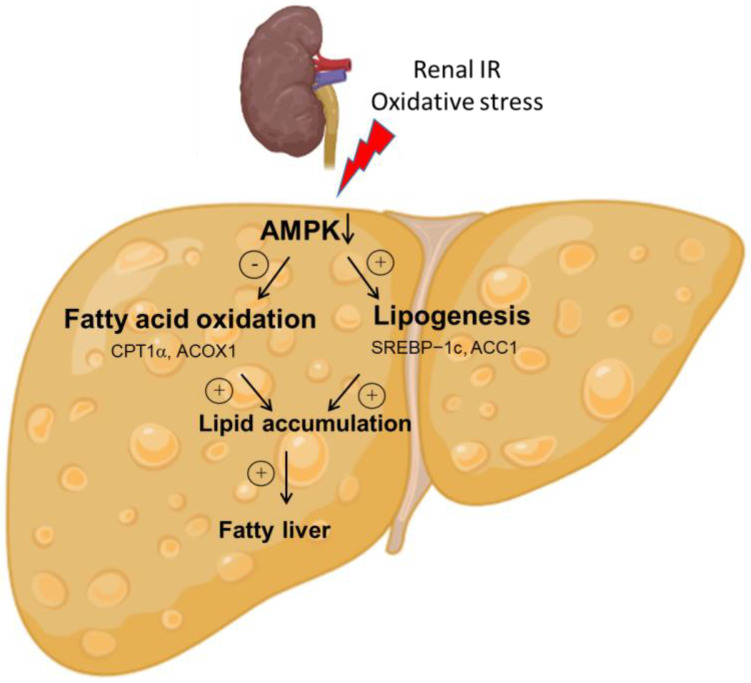
Graphical illustration and proposed mechanism of AKI-induced hepatic lipid accumulation. In rats with ischemia-refusion-induced AKI, inhibition of hepatic AMPK signaling attenuated SREBP-1c-mediated lipogenesis and increased fatty acid oxidation identified by elevated expression of CPTIα and ACOX1. Decreased fatty acid synthesis and increased lipogenesis caused hepatic lipid accumulation after renal IR. The circled + and − symbols stand for stimulation and inhibition, respectively. Abbreviations: AMPK = AMP-activated protein kinase; SREBP-1c = sterol regulatory element-binding transcription factor 1c; ACC1 = acetyl-CoA carboxylase 1; CPTIα = carnitine palmitoyltransferase-I-alpha; ACOX1 = acyl-CoA oxidase1.

The liver is one of the major organs affected in both AKI patients and experimental IR animals. In this study, we found that AKI-induced oxidative stress contributed to the inhibition of AMPK-mediated lipid metabolism, leading to hepatic lipid accumulation (Figure 6). Our data demonstrated that renal IR increased both plasma and liver MDA, impaired liver function and caused hepatic lipid accumulation. We have also demonstrated that AMPK signaling was downregulated after renal IR, significantly altering the expression of genes involved in lipogenesis and fatty acid oxidation.

AMPK, a critical sensor of cellular energy status, plays an important role in energy production and mitochondrial homeostasis [36]. This kinase can be activated by cellular stresses involving ATP depletion and AMP elevation [37]. Ma and colleagues [38] showed a decreased ATP production in the kidney during the ischemic phase due to oxygen depletion and, consequently, the increased AMP to ATP ratio activated AMPK. However, the AMPK in the kidney was inhibited during the reperfusion phase [38]. In our current study, the phosphorylated AMPK was decreased in the liver of rats after renal IR. This was accompanied by the increased expression of SREBP-1c and ACC1, which regulate lipogenesis. SREBP-1c plays a crucial role as a transcription factor in the regulation of lipogenesis by upregulating the expression of key enzymes responsible for this process, such as ACC1, which is the rate-limiting enzyme of lipogenesis. AMPK exerts its effects by phosphorylating SREBP-1c, which hinders the proteolytic cleavages and nuclear translocation of SREBP-1c. This, in turn, inhibits hepatic lipogenesis [39]. In addition to lipogenesis, promoting lipid consumption by mitochondrial fatty acid oxidation is also essential for reducing lipid levels [40]. The rate-limiting step of mitochondrial fatty acid oxidation is regulated by CPTIα [41], while ACOX1 catalyzes the rate-limiting reaction of peroxisomal fatty acid oxidation [42]. Our findings demonstrated a significant reduction in the expression of both CPTIα and ACOX1 in the liver following renal IR, indicating a decrease in fatty acid oxidation. Previous preclinical studies have shown that activation of AMPK can decrease hepatic lipid content [17,25,43]. With its ability to increase fatty acid oxidation and inhibit lipogenesis in the liver, AMPK is a therapeutic target for the treatment of NAFLD [44]. Collectively, our results suggest that the inhibition of AMPK may contribute to the increased accumulation of hepatic lipids observed in renal IR-induced AKI, as illustrated in Figure 6. To be able to expand the therapeutic arsenal for managing renal IR-induced AKI and associated hepatic lipid accumulation, clinical studies to evaluate the efficacy of AMPK activation are essential.

AKI is widely recognized to induce oxidative stress at both local and systemic levels. Our previous study demonstrated that renal IR caused a marked decrease in glutathione levels in the kidney and liver [10]. Glutathione is a major endogenous nonenzymatic antioxidant, and its depletion can compromise the ability of the body to cope with oxidative stress [10]. In the present study, the elevation of oxidative stress biomarkers (lipid peroxides) was detected in both the plasma and the liver. Consistent with our animal data, the current in vitro experiments showed that the oxidative stress inducer H_2_O_2_ inhibited AMPK phosphorylation and altered the expression of its target genes in hepatocytes. This, in turn, resulted in intracellular lipid accumulation. Previous studies have shown that ROS could inhibit AMPK activity in cardiomyocytes and hepatocytes, as well as during energy starvation in cardiomyocytes [45,46,47]. The inhibition of AMPK signaling blocked the ROS-NFκB signal cascade, resulting in the attenuation of endotoxemia-induced liver injury [48]. However, a previous study showed that AMPK was activated directly by H_2_O_2_ in human embryonic kidney cells through oxidative modification of the AMPKα subunit [49]. These discrepancies could arise from differences in experimental conditions and the variations in the levels of ROS scavenging systems in the cell types used [47,50]. Multiple studies have shown that there is a complex interplay between AMPK activity and ROS production. On the one hand, excessive ROS production has been shown to inhibit AMPK activity through various mechanisms, such as direct oxidation of AMPK subunits and disruption of cellular redox balance [13,36]. On the other hand, AMPK activation diminishes oxidative stress in endothelial cells [51]. Therefore, targeting the AMPK pathway may provide new therapeutic opportunities for treating diseases related to oxidative stress and dysregulated lipid metabolism.

The liver plays a crucial role in lipid metabolism by managing the uptake, synthesis, and metabolism of fatty acids, as well as regulating the export and redistribution of lipids. Ischemia-reperfusion injury can result in a large influx of ROS during reperfusion, leading to tissue damage and complications in organ transplantation, stroke, and myocardial infarction [52,53]. Animal studies have demonstrated that compounds that activate AMPK, such as berberine and quercetin, can improve NAFLD and ameliorate IR injury in an AMPK-dependent manner [44,54,55]. These findings suggested that natural compounds that modulate AMPK activity may serve as promising therapeutic agents for treating AKI. Owing to the crosstalk of distant organ injury, an improvement in AKI may pause the worsening liver conditions observed in NAFLD. In contrast, up to 70% of patients with acute liver failure developed AKI, and among those patients, 30% received renal replacement therapy [56]. Although the liver is an important organ responsible for lipid metabolism, adipose tissue also plays significant roles in body homeostasis by storing lipids as an energy source and releasing adipokine to regulate metabolic functions [57]. However, the impact of AKI on adipocyte lipid metabolism remains unknown. The strengths and weaknesses of the current study should be considered. While our study is the first to suggest that renal IR-induced oxidative stress inhibits AMPK, leading to lipid accumulation in the liver, the underlying mechanisms require further investigation. Previous research has shown that liver-specific AMPK knockout exacerbates liver lipid accumulation, steatosis, and inflammation in mouse models of obesity and NAFLD [58,59], supporting the potential of AMPK as a therapeutic target for AKI-induced hepatic lipid accumulation. Further research is warranted to study the long-term effects of AKI on hepatic lipid metabolism and clinical outcomes.

## 5. Conclusions

In conclusion, the present study showed that oxidative stress induced by renal IR inhibited AMPK signaling, leading to lipid accumulation (Figure 6). This, in turn, may result in increased hepatic lipid accumulation during AKI due to decreased fatty acid metabolism and increased lipogenesis. Further research is needed to investigate the long-term effect of AKI on hepatic lipid accumulation and its impact on clinical outcomes. By gaining a better understanding of the responses of both local and distant organs to AKI, new therapeutic targets may be identified for patients with multiple organ injuries.

## Figures and Tables

**Table 1:** Gene primer sequences of rats and humans used for real-time PCR.

Target Gene	Forward Primer (5′−3′)	Reverse Primer (5′−3′)	Size (bp)	Accession Number
Rat				
*SREBP*-*1c*	GGCCCTGTGTGTACTGGTCT	AGCATCAGAGGGAGTGAGGA	88	NM_001276708.1
*ACOX1*	CTGATGAAATACGCCCAGGT	GGTCCCATACGTCAGCTTGT	75	NM_001414015.1
*ACC1*	TGAGGAGGACCGCATTTATC	AAGCTTCCTTCGTGACCAGA	221	NM_022193.2
*CPTIα*	CAGCTCGCACATTACAAGGA	TGCACAAAGTTGCAGGACTC	128	XM_039102321.1
*β-actin*	ACAACCTTCTTGCAGCTCCTC	GACCCATACCCACCATCACA	198	NM_031144.3
Human				
*SREBP*-*1c*	ACACAGCAACCAGAAACTCAAG	AGTGTGTCCTCCACCTCAGTCT	153	NM_001005291.3
*ACOX1*	GGCGCATACATGAAGGAGACCT	AGGTGAAAGCCTTCAGTCCAGC	112	NM_001185039.2
*ACC1*	TTCACTCCACCTTGTCAGCGGA	GTCAGAGAAGCAGCCCATCACT	99	XM_047435894.1
*CPTIα*	CGATGTTACGACAGGTGGTTTGACA	AGTGCCCATCCTCCGCATAG	172	NM_001876.4
*β-actin*	AGATCAAGATCATTGCTCCTCCT	GATCCACATCTGCTGGAAGG	95	NM_001101.5

## Data Availability

Data are contained within the article.
